# Verifying the Japanese Version of Pediatric Delirium and Withdrawal Syndrome Assessment Scale: SOS-PD Validation Study for Iatrogenic Withdrawal Syndrome

**DOI:** 10.3390/children12030372

**Published:** 2025-03-17

**Authors:** Yujiro Matsuishi, Haruhiko Hoshino, Yuki Enomoto, Takahiro Kido, Nobutake Shimojo, Bryan J. Mathis, Erwin Ista, Yoshiaki Inoue

**Affiliations:** 1 Adult and Elderly Nursing, Faculty of Nursing, Tokyo University of Information Science, Chiba 265-8501, Japan; 2 Department of Nursing, Faculty of Medical Technology, Teikyo University, Tokyo 113-8510, Japan; 3 Department of Emergency and Critical Care Medicine, Faculty of Medicine, University of Tsukuba, Tsukuba 305-8577, Japanyinoue@md.tsukuba.ac.jp (Y.I.); 4 Department of Pediatrics, University of Tsukuba Hospital, Tsukuba 305-8577, Japan; 5 Department of Cardiovascular Surgery, Institute of Medicine, University of Tsukuba, Tsukuba 305-8577, Japan; 6 Department of Neonatal and Pediatric Intensive Care, Division of Pediatric Intensive Care, Erasmus University Medical Center-Sophia Children’s Hospital, 3015 GD Rotterdam, The Netherlands

**Keywords:** iatrogenic withdrawal syndrome, pediatrics, intensive care unit, validation study, Sophia Observation withdrawal Symptoms-Paediatric Delirium scale, Japanese version, reliability, validity

## Abstract

**Background:** Iatrogenic withdrawal syndrome (IWS) poses a significant clinical challenge in pediatric intensive care units (PICUs) within Japan. Despite the existing availability of tools to assess pain and delirium, a validated instrument specifically designed for IWS has been notably absent in Japanese clinical practice. The Sophia Observation withdrawal Symptoms-Paediatric Delirium (SOS-PD) scale is globally recognized as an effective tool for IWS evaluation. To bridge this gap, this study aimed to validate the Japanese version of the SOS-PD scale. **Methods**: A prospective, cohort, observational study was undertaken in a single-center PICU in Japan. Participants ranged from neonates to children aged 20 years, excluding those with pre-existing neurological conditions or coma. Criterion validity was evaluated by comparing Japanese SOS-PD scale scores between a Weaning Group (WEAN) undergoing sedative/opioid tapering and a Maintenance Group (MAIN) receiving stable medication. Correlation analysis was also conducted against pediatric intensivists’ observational NRS (NRSobs). Inter-rater reliability of the Japanese SOS-PD scale was assessed utilizing kappa statistics and intraclass correlation coefficient (ICC). **Results**: In support of criterion validity, the WEAN group demonstrated significantly higher scores in both NRSobs and the IWS component of the Japanese SOS-PD scale compared to the MAIN group (*p* < 0.001). A strong correlation was observed between the Japanese SOS-PD IWS component and NRSobs (r = 0.91, *p* < 0.001). Inter-rater reliability was also robust, with a kappa coefficient of 0.95 and an ICC of 0.98. **Conclusions**: The Japanese version of the SOS-PD scale exhibits strong validity and inter-rater reliability for IWS assessment within Japanese PICUs. This validated instrument can support the early detection and appropriate management of pediatric IWS in Japan, with the potential to enhance the quality of patient care.

## 1. Introduction

Iatrogenic Withdrawal Syndrome (IWS) represents a notable clinical challenge within pediatric intensive care units (PICUs), particularly among children subjected to prolonged administration of opioids and benzodiazepines. The prevalence of IWS in critically ill pediatric patients varies widely. The reported incidence of IWS in critically ill pediatric patients varies widely, with reports indicating that 57% of PICU patients and 60% of PICUs are affected [1,2,3,4], underscoring diagnostic challenges and the need for standardized assessment tools. IWS arises after a sudden reduction or cessation of sedative or analgesic medications, leading to a constellation of withdrawal symptoms [5], including tremors, agitation, sleeplessness, inconsolable crying, diarrhea, and sweating [5]. The severity and presentation of symptoms can vary, making early detection and management crucial to mitigate adverse outcomes [4].

Several assessment tools have been developed to facilitate the identification of IWS in pediatric populations [5,6,7]. Among these, the Sophia Observation withdrawal Symptoms-Paediatric Delirium (SOS-PD) scale is recognized as an effective tool for assessing IWS. The SOS-PD scale evaluates 22 symptoms, of which 15 are specific to IWS, allowing healthcare professionals to accurately identify withdrawal symptoms in pediatric patients. [8] Additionally, SOS-PD has the advantage of simultaneously assessing pediatric delirium, making it a versatile and user-friendly tool in clinical practice [8].

Currently, pain and delirium assessment scales have already been validated, but there is no validated tool specifically for iatrogenic withdrawal syndrome (IWS) in Japan. Therefore, translating the SOS-PD scale into Japanese offers significant clinical benefits. Introducing a standardized assessment tool into Japanese healthcare settings can facilitate early detection and appropriate management of pediatric IWS, potentially improving the quality of patient care.

This study aims to validate the Japanese version of the SOS-PD scale to ensure its accuracy, reliability, and effectiveness in identifying and managing pediatric IWS within Japanese healthcare settings.

## 2. Method

### 2.1. Study Design

This study was conducted in a pediatric intensive care unit as a prospective cohort observational clinimetric study.

### 2.2. Patient Selection

Between October 2018 and March 2020, we enrolled patients in the mixed-use (eight-bed post-surgical and internal medicine) pediatric ICU at the University of Tsukuba Hospital (Tsukuba, Japan) on Tuesdays at 2 p.m. or 6 p.m. The inclusion criteria were newborns with a gestational age (GA) exceeding 40 weeks and children up to 20 years of age, regardless of their sedation status. Sedation levels were recorded using the Richmond Agitation-Sedation Scale (RASS), and the percentage of patients in each category will be presented. RASS assessments were performed under routine sedation. Patients were excluded if they had neurological abnormalities (e.g., traumatic brain injury, encephalitis) or were in a coma, defined as a score below—4 on RASS.

### 2.3. Group Division Based on Weaning Status

Patients who received continuous administration of opioids or benzodiazepines were categorized based on their weaning status. The date of initiation of drug reduction was used as the reference point, and the weaning group was defined as those who were weaned off within 72 h from the last administration.

### 2.4. Data Collection

Data collection tools included the Japanese version of the Sophia Observation Withdrawal Symptoms-Paediatric Delirium (SOS-PD) scale and the observational NRS scale (NRSobs). Patient assessments using the SOS-PD scale and NRSobs were conducted weekly, specifically on Wednesdays at 1:00 p.m., during the weekly rounds with physicians and researchers. This weekly evaluation timing was chosen because, while the SOS-PD scale recommends more frequent assessments such as once per shift, when delirium is suspected, or 2–4 h post-intervention for delirium treatment, our study aimed to observe changes in patient status over more extended intervals.

We hypothesized that weekly assessments would be sufficient to detect meaningful changes in IWS symptoms, as observing patients with a shorter interval might capture similar clinical status from the last observation in our PICU setting.

Two Researchers administered the Japanese version of the SOS-PD scale, while two pediatric intensivists evaluated the severity of IWS using the NRSobs. Demographic data and patient characteristics were extracted from medical records.

### 2.5. Data Analysis

The data analysis aimed to validate the Japanese version of the SOS-PD scale for assessing IWS in the PICU setting. Therefore, we report IWS component score of the SOS-PD scale only. The analysis focused on evaluating the criterion validity and interobserver reliability of the Japanese version of the SOS-PD scale. Criterion validity was assessed by comparing SOS-PD scale scores and NRSobs scores between the WEAN and MAIN groups using statistical tests and correlation analysis. Interobserver reliability was examined by calculating weighted kappa coefficients and intraclass correlation coefficients (ICC) to measure the agreement between researchers’ assessments. Detailed descriptions of the specific statistical methods are provided in the Section 2.9 below.

### 2.6. Severity of IWS

In this study, the “severity of IWS” was operationally defined as the assessment by pediatric intensivists using the NRSobs. The NRSobs is a scale designed to intuitively assess the severity of IWS using a numerical range from 0 (indicating no IWS symptoms) to 10 (representing the most severe IWS symptoms). Pediatric intensivists, blinded to the SOS-PD scale scores, evaluated patients based on their clinical judgment and experience, utilizing the NRSobs to represent the overall severity of IWS. The NRSobs assessment was performed concurrently with the SOS-PD scale assessment, during the weekly rounds conducted every Wednesday at 1:00 p.m.

Therefore, the NRSobs data collection also followed the same weekly schedule and timing as described for the SOS-PD scale in the Section 2.4.

### 2.7. Measurement Properties

#### Criterion Validation of IWS

In this study, to verify whether the Japanese version of the SOS-PD scale IWS items are consistent with clinically valid IWS assessments, we referenced the criterion validity evaluation in the COSMIN (COnsensus-based Standards for the selection of health Measurement Instruments) guideline [9]. In the COSMIN guideline, criterion validity evaluates the extent to which a measurement tool under evaluation aligns with a criterion considered to be a “gold standard”. In this study, we adopted the observational NRS (Numerical Rating Scale) scale by pediatric intensivists as a surrogate criterion for IWS assessment in clinical settings, based on evidence from existing literature of its established use in evaluating IWS severity [5,9,10,11]. By comparing the scores of the Japanese version of the SOS-PD scale IWS items with NRSobs scores, we validated the criterion validity of the Japanese version of the SOS-PD scale. This approach aligns with the criterion validity evaluation approach recommended by the COSMIN guideline.

### 2.8. Interobserver Reliability of IWS (Iatrogenic Withdrawal Syndrome)

To verify whether the Japanese version of the SOS-PD scale IWS items can be consistently evaluated by multiple raters, we focused on evaluating the reliability, specifically interobserver reliability, in the COSMIN guideline. In the COSMIN guideline, reliability is a concept indicating the extent of measurement error, and interobserver reliability indicates the extent to which assessments agree when different raters evaluate the same subject. In this study, two researchers (Y.M., H.H.) with experience using withdrawal symptom scales for over three years in prior studies on pediatric delirium, sedation, and pain in Japanese settings (Japanese version) independently evaluated the same patients, and by statistically verifying the agreement of their assessments, we evaluated the interobserver reliability of the Japanese version of the SOS-PD scale. This methodology aligns with the reliability assessment methods recommended by the COSMIN guideline.

### 2.9. Statistical Analysis

#### 2.9.1. Criterion Validation—Statistical Analysis

To verify the criterion validity of IWS, we compared the NRSobs scores and Japanese version of the SOS-PD scale IWS item scores between the weaning group (WEAN) and the maintain group (MAIN). For group comparisons, we used *t*-test, setting the significance level at *p* < 0.05. Furthermore, to evaluate the correlation between the Japanese version of the SOS-PD scale IWS items and NRSobs, we used Spearman’s rank correlation coefficient to calculate the correlation coefficient (r). The interpretation of the correlation coefficient was based on Guilford’s Rule of Thumb [12]. This correlation analysis aims to evaluate the construct validity (convergent validity) of the Japanese version of the SOS-PD scale.

#### 2.9.2. Interobserver Reliability—Statistical Analysis

To evaluate the interobserver reliability of the Japanese version of the SOS-PD scale IWS items, we calculated the agreement between researchers using the weighted kappa coefficient (κ) [13] and the intraclass correlation coefficient (ICC) for average measures with a two-way random effects model [14]. For the calculation of the kappa coefficient and ICC, we used and appended the 95% confidence interval. For statistical analysis, we used R software(version 4.4.3).

### 2.10. Sample Size Calculation

Sample size was calculated based on the prevalence of IWS. While COSMIN guidelines recommend sample size considerations based on the specific measurement property being evaluated, for validation studies, particularly in contexts where the prevalence of the condition is a key factor for tool implementation, prevalence-based calculations can be informative. Based on our previous report, we assumed the prevalence of IWS to be 52%. We determined that a sample size based on the epiR package and 136 observations would be required for a significance level (α) of 0.05 and test power (1-β) of 0.80. While COSMIN guidelines suggest a minimum of 100 participants for reliability and criterion validity studies, our prevalence-based calculation led to a similar sample size, ensuring we could adequately capture a sufficient number of IWS cases within our study population to robustly evaluate the Japanese version of the SOS-PD scale in our clinical setting.

### 2.11. Ethics

This study was carried out under laws equivalent to or derived from the principles of the Declaration of Helsinki and was approved by the University of Tsukuba Institutional Review Board (approval # H28–085). In addition, this study was conducted under the approval of the Ethics Committee and was performed using a caregiver opt-out format. In this format, caregivers of newborns were provided with comprehensive information about the study and given the opportunity to decline participation on behalf of their newborn. This approach is ethically appropriate for research involving newborns, as newborns themselves are unable to provide or decline informed consent for study participation.

## 3. Result

### 3.1. Patient Demographics and Characteristics

From October 2018 to April 2020, we initially collected 271 observations. Among these, 135 observations were excluded based on the following exclusion criteria: 53 observations from patients in a coma, 76 observations from patients with brain dysfunction, and 6 observations from patients aged over 20 years old or newborns with a gestational age less than 40 weeks at the time of observation. After applying these exclusion criteria, 136 observations remained and were included in the validity and reliability analyses. These 136 observations were obtained from 75 unique patients, as some patients contributed multiple observations during their PICU stay. The median number of assessments per patient was 1 (IQR 1). Therefore, we evaluated a total of 136 observations from 75 patients for this study.

Table 1 presents the baseline demographic characteristics of the 75 unique patients enrolled in our study.

Regarding age, patients were on average 9 months old, with a standard deviation of 12.2 months. This standard deviation, being larger than the mean, indicates a considerable variability in the ages of the patients included, ranging from neonates to older children within the pediatric age range. This heterogeneity in age is an important characteristic of our study population and reflects the diverse pediatric patients typically admitted to a PICU setting.

The gender distribution shows that females slightly outnumbered males, accounting for 57% (n = 43) of the participants. Analyzing the primary diagnoses, cardiac surgical cases constituted the majority, representing 68% of the patient cohort (n = 51). This high proportion of cardiac surgical patients is characteristic of the patient population in our PICU, which is a mixed-use unit that includes post-surgical patients. Medical cases represented the second largest diagnostic group at 22% (n = 17), followed by abdominal surgical cases at 8% (n = 6) and a small proportion of thoracic surgical cases at 1% (n = 1). This distribution of diagnoses highlights the spectrum of critical illnesses managed within our PICU.

Pre-existing conditions were also noted. Trisomy 21 (Down syndrome) was present in 12% of the patients (n = 9), and developmental delay was observed in 25% of the cohort (n = 19). The presence of these conditions is clinically relevant as they may potentially influence the presentation of withdrawal symptoms and the interpretation of assessment scales in this patient population.

Finally, considering respiratory support at the time of enrollment, approximately 30% of the patients (n = 23) were receiving mechanical ventilation. This indicates that a significant portion of our study population consisted of critically ill patients requiring advanced respiratory support, further emphasizing the clinical importance of IWS assessment in this setting. The remaining 70% (n = 52) were evaluated while not receiving mechanical ventilation, suggesting the SOS-PD scale was assessed across a range of respiratory support levels within the PICU.

Table 2 presents the baseline characteristics of the research evaluation units. Participants had an average age of 9 months (±12.2 months) and 57% (n = 78) were female. Regarding the diagnosis categories, 76% (n = 104) of the cases were cardiac surgical, followed by 16% (n = 23) medical cases, 5% (n = 7) abdominal surgical cases, and 1% (n = 3) thoracic surgical cases. Additionally, 18% (n = 25) of participants had trisomy and 30% (n = 41) had developmental delays. At the time of observation, 30% (n = 41) of patients were receiving mechanical ventilation (MV), 16% (n = 22) were on non-invasive positive pressure ventilation (NPPV), and 10% (n = 14) were using nasal high-flow (NHF). Sedation or opioid weaning status revealed that 66% (n = 90) belonged to the control group, while 33% (n = 46) were in the weaning group.

In our study, the prevalence of delirium was 30% (41/136 observations) during the total observation period. Interestingly, the prevalence of delirium tended to be numerically higher in non-mechanically ventilated patients (58%, n = 24) compared to mechanically ventilated patients (41%, n = 17); however, there was no statistically significant association between mechanical ventilation and the presence of delirium (*p* = 0.06).

### 3.2. Severity of IWS

**Figure 1:**
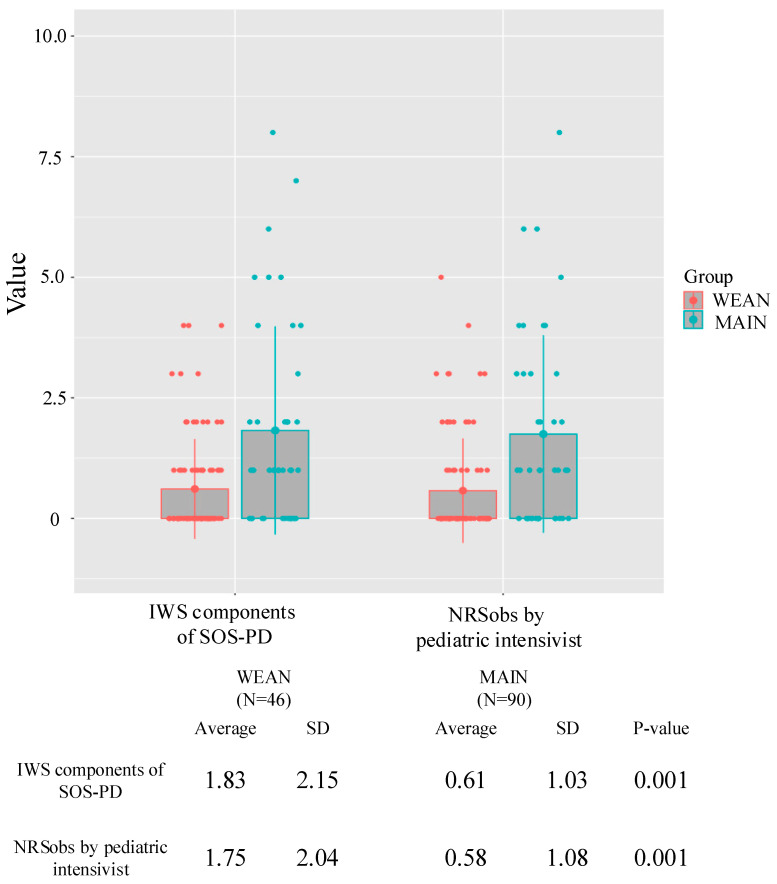
Severity of IWS. This figure provides a detailed comparison of IWS severity between the WEAN and MAIN groups. The data, presented as mean and standard deviation (SD), illustrate the scores for both the IWS components of the SOS-PD scale and the NRSobs by pediatric intensivists. The *p*-values (0.001 for both comparisons) indicate statistically significant differences between the groups for both measures of IWS severity.

Figure 1 shows the severity of IWS during the total observation period and the severity of IWS in each group. The mean NRSobs score in the WEAN group was 1.75 (SD 2.04), while it was 0.58 (SD 1.08) in the MAIN group. This difference was statistically significant (*p* = 0.001).

Similarly, the SOS-PD IWS component scores also showed a significantly greater severity of IWS in the WEAN group compared to the MAIN group. Specifically, the mean SOS-PD IWS component score was 1.83 (SD 2.15) in the WEAN group, and 0.61 (SD 1.03) in the MAIN group. This difference was also statistically significant (*p* = 0.001) (Figure 1).

### 3.3. Criterion Validation of IWS

**Figure 2:**
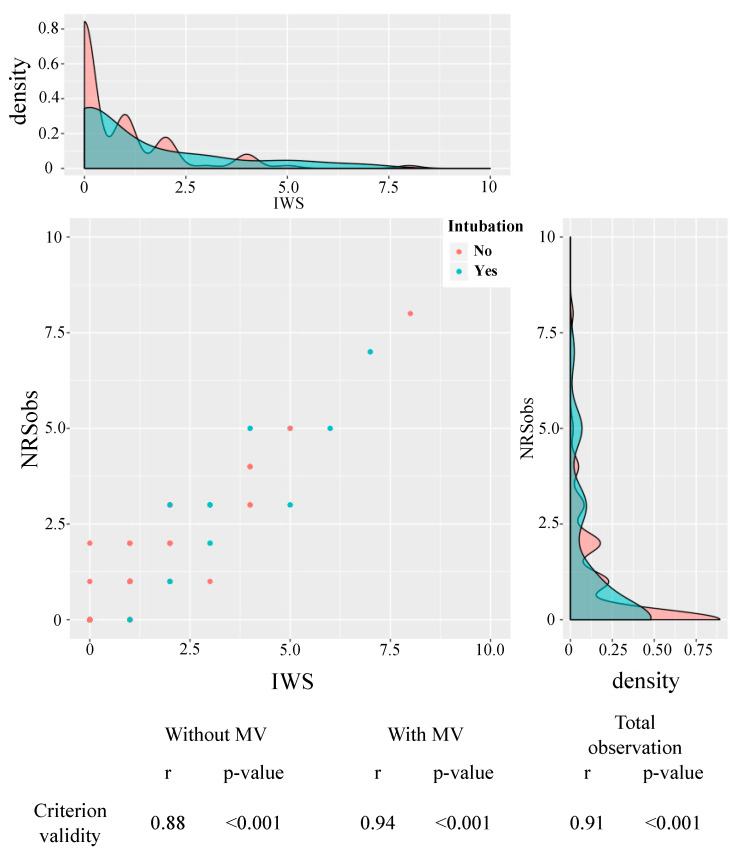
Criterion Validity of IWS. This figure presents the criterion validity results for the Japanese version of the SOS-PD scale IWS component. Criterion validity is assessed by the correlation (r) between the Japanese SOS-PD scale and the pediatric intensivists’ NRSobs. The figure displays Criterion validity, NRSobs density, and NRSobs mean (NRSobs) across three groups: Without MV, With MV, and Total observation. High correlations were observed in both the MV-managed group (r = 0.94, *p* < 0.001) and the non-MV-managed group (r = 0.88, *p* < 0.001).

The agreement between NRSobs by pediatric intensivists and Japanese SOS-PD IWS components by researchers was highly correlated in the total observations (r = 0.91, *p* < 0.001). This result suggests that the Japanese SOS-PD IWS component has high criterion validity when compared to NRSobs, which is considered a clinically valid IWS assessment. Furthermore, high correlations were observed in both the MV-managed group (r = 0.94, *p* < 0.001) and the non-MV-managed group (r = 0.88, *p* < 0.001) (Figure 2).

### 3.4. Interobserver Reliability

The high reliability of the Japanese SOS-PD IWS between researchers is shown in Table 3 (κ = 0.95, 95%CI [0.92–0.98], ICC = 0.98, 95%CI [0.97–0.98]). This trend did not differ between MV (κ = 0.96, 95%CI [0.92–1.0], ICC = 0.98, 95%CI [0.97–0.99]) and non-MV patients (κ = 0.93, 95%CI [0.88–0.97], ICC = 0.97, 95%CI [0.96–0.98]). The current results strongly suggest that the Japanese SOS-PD IWS can be assessed with extremely high consistency between researchers. Similarly high interobserver reliability was confirmed in both MV-managed and non-MV-managed patients.

## 4. Discussion

This study aimed to validate the Japanese version of SOS-PD Scale, an important tool for assessing IWS in PICUs. The findings demonstrated that this tool is effective in detecting IWS early in PICU settings.

The primary contribution of this study is the validation of the Japanese SOS-PD scale, which demonstrated high criterion validity and interobserver reliability. Specifically, the SOS-PD scale showed a very high correlation with the widely used observational tool for pediatric delirium and IWS, NRSobs (r = 0.91, *p* < 0.001), supporting the usefulness of the SOS-PD scale for early diagnosis. Ista et al. emphasized the importance of standardized tools for assessing IWS and the results of this study are consistent with that finding [8].

Additionally, the interobserver reliability (κ = 0.95, ICC = 0.98) indicating strong score consistency between the two researchers in this study. However, this finding has limitations in generalizability. This agreement was observed specifically between two highly experienced researchers. Clinical practice involves diverse staff with varied experience, and it is uncertain if such high reliability would extend to all users in routine care. Future research should assess interobserver reliability across a broader range of clinicians to fully evaluate the scale’s practical utility and generalizability in Japanese PICUs. This broader assessment is essential to confirm the scale’s robustness and widespread applicability.

The clinical importance of the SOS-PD scale cannot be overstated. IWS is a common issue in pediatric critical care, especially in patients who have received prolonged sedation or analgesia. These symptoms can manifest as acute anxiety, tremors, and restlessness, which, if not identified and managed early, may lead to prolonged recovery times and poor outcomes [15,16]. Using the SOS-PD scale to accurately assess and monitor these symptoms is crucial for preventing complications and ensuring that appropriate interventions, such as tapering sedative medications, are implemented early. Moreover, this study demonstrated that the SOS-PD scale is effective for both ventilated and non-ventilated patients. This means the tool is applicable to a wide range of pediatric patients in PICUs and, as a previous study pointed out [17], pediatric patients on mechanical ventilation are particularly vulnerable to IWS due to prolonged sedation and analgesia. Therefore, the SOS-PD scale is a versatile tool that can be used for various patient groups in critical care settings. While the SOS-PD scale is a valuable tool, other assessment tools for IWS exist, such as the Withdrawal Assessment Tool-1 (WAT-1) [5]. The WAT-1 is another validated and commonly used scale for assessing withdrawal symptoms in pediatric patients. Both SOS-PD and WAT-1 are important tools in the management of IWS, and the validation of the Japanese version of SOS-PD in this study provides clinicians in Japanese PICUs with another robust option for IWS assessment, particularly considering SOS-PD’s added capability to simultaneously assess delirium, which is also crucial in this patient population.

There are several limitations to this study and future research must address these challenges. Since the study was conducted at a single institution with a relatively homogeneous patient population, the generalizability of the findings may be limited. Previous studies highlighted the importance of a standardized approach to delirium screening tools in pediatric ICUs [8,18] and future studies should involve multiple institutions with diverse patient populations to confirm the scale’s applicability and universality in Japan. Research on Post Intensive Care Syndrome (PICS) has begun to enable measurements in Japan [19,20] and the importance of studies related to pediatric PICS (PICS-p) [21] is also gaining recognition. Moreover, expanding the applicability of the SOS-PD scale beyond pediatric ICUs to other healthcare settings is necessary. For example, it could be adapted for use in general wards or outpatient settings, which would further broaden its impact in early detection and management of IWS.

## 5. Limitation

This study has several limitations that should be considered when interpreting the results. These include the single-center design, the relatively homogeneous patient population, and the fact that interobserver reliability was assessed only between two highly experienced researchers. These limitations are common to validation studies conducted in single centers and should be addressed in future multi-center research. 

A specific limitation of this study is the inclusion of patients with Down syndrome and developmental delays. While SOS-PD exclusion criteria include severely disturbed behavioral pattern due to neurological disease, our exclusion criteria focused on explicit neurological abnormalities or coma. This aligns with SOS-PD’s intent to exclude behavior primarily driven by neurological conditions, not iatrogenic withdrawal.

Patients with Down syndrome and developmental delays were included as they didn’t necessarily have severely disturbed behavior from their conditions at assessment. However, their presence might influence SOS-PD item presentation, potentially limiting generalizability, especially to those with pronounced behavioral challenges. Future research should specifically validate SOS-PD in pediatric Down syndrome and developmental delay populations, considering neurologically-driven behavioral disturbances. This would clarify the scale’s utility across diverse PICU pediatric populations.

Another potential limitation arises from the relatively low average SOS-PD IWS component score (1.8) observed in our study. This low average score, while indicating a generally low severity of IWS in our cohort, might be attributable to the intensive clinical attention and proactive management of withdrawal symptoms that are standard practice in our PICU. Our clinical practice emphasizes careful monitoring and preemptive interventions to minimize iatrogenic withdrawal syndrome in at-risk pediatric patients. As this study is observational in nature, the low average score may reflect the effectiveness of our clinical practices in mitigating IWS, rather than the inherent limitations of the SOS-PD scale itself. Therefore, the generalizability of our findings regarding the prevalence and severity of IWS, particularly the low average score, to PICUs with less stringent withdrawal management protocols may be limited. Future studies in diverse PICU settings with varying approaches to withdrawal management are needed to further explore the range of SOS-PD scores and the scale’s performance across different clinical contexts.

## 6. Conclusions

The validation of the Japanese SOS-PD scale for assessing IWS represents a significant advancement in pediatric critical care. This tool has demonstrated high validity and reliability.

## Figures and Tables

**Table 1:** Baseline patient characteristics.

Variable	Participants N = 75
Age (months) ± SD	9 ± 12.2
Female n (%)	43 (57)
Diagnosis	
Cardiac Surgical, n (%)	51 (68)
Medical, n (%)	17 (22)
Abdominal Surgical, n (%)	6 (8)
Thoracic Surgical, n (%)	1 (1)
Trisomy Patients, n (%)	9 (12)
Developmental Delay, n (%)	19 (25)
Mechanical Ventilation	23 (30)

SD = standard deviation.

**Table 2:** Baseline characteristics of research evaluation units.

Variable	Total Observation N = 136
Age (months) ± SD	7 ± 10.2
Female n (%)	78 (57)
Diagnosis	
Cardiac Surgical, n (%)	104 (76)
Medical, n (%)	23 (16)
Abdominal Surgical, n (%)	7 (5)
Thoracic Surgical, n (%)	3 (1)
Trisomy Patients, n (%)	25 (18)
Developmental Delay, n (%)	41 (30)
Ventilation at Observation	
Mechanical Ventilation (MV)	41 (30)
Non-invasive Positive Pressure Ventilation (NPPV)	22 (16)
Nasal High Flow (NHF)	14 (10)
Weaning of Sedation/Opioid Status at Observation	
Control Group	90 (66)
Weaning Group	46 (33)
Prevalence of Delirium Within Total Observations Total With MV Without MV	41 (30) 17 (41) 24 (58)

SD = standard deviation.

**Table 3:** Reliability of Japanese Version of SOS-PD IWS.

Variable	Statistics	Without MV N = 90	With MV N = 35	Total Number of Observations N = 136
IWS Components of SOS-PD	Weighted kappa ^a^	0.94 [0.90–0.98]	0.96 [0.92–1.0]	0.95 [0.93–0.98]
	ICC ^b^	0.97 [0.96–0.98]	0.98 [0.97–0.99]	0.98 [0.97–0.98]

^a:^ Data are kappa coefficients [95% confidence interval]. ^b:^ Data are Intra class correlation coefficients [95% confidence interval].

## Data Availability

The datasets generated and/or analyzed during the current study are not publicly available due to conducting the sub-analysis but are available from the corresponding author upon reasonable request. Researchers interested in accessing the data for future studies may contact Dr. Yujiro Matsuishi, the co-corresponding author, to inquire about data availability.
